# Supplementary material for: Transcription recycling assays identify PAF1 as a driver for RNA Pol II recycling
Source: Nat Commun. 2021 Nov 3;12:6318. doi: 10.1038/s41467-021-26604-1 (PMC8566496; doi:10.1038/s41467-021-26604-1)
Supplement: Supplementary file 1 — Supplementary Information [file 41467_2021_26604_MOESM1_ESM.pdf]

Transcription recycling assays identify PAF1 as a driver for RNA Pol II recycling

Zhong Chen✉, William Hankey, Yue Zhao, Jeff Groth, Furong Huang, Hongyan Wang, Alexandre Rosa Campos, Jiaoti Huang, Robert G. Roeder & Qianben Wang✉

Supplementary Information

SUPPLEMENTARY FIGURES

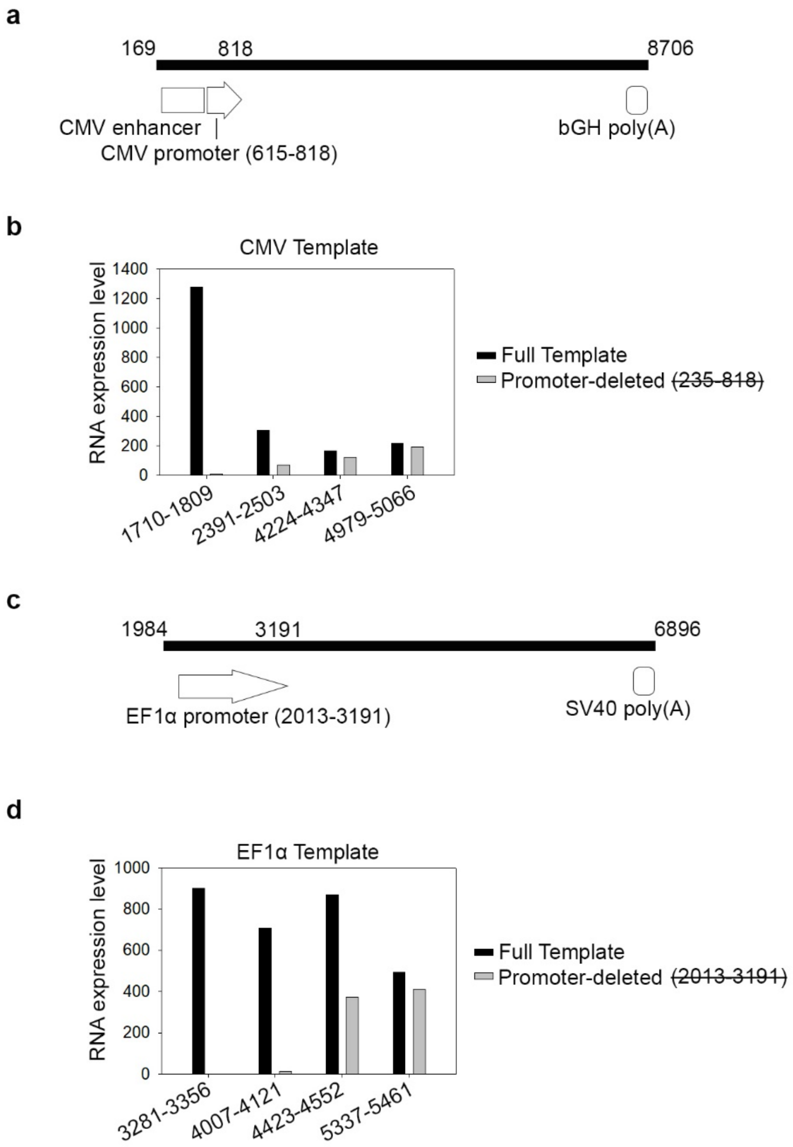

e

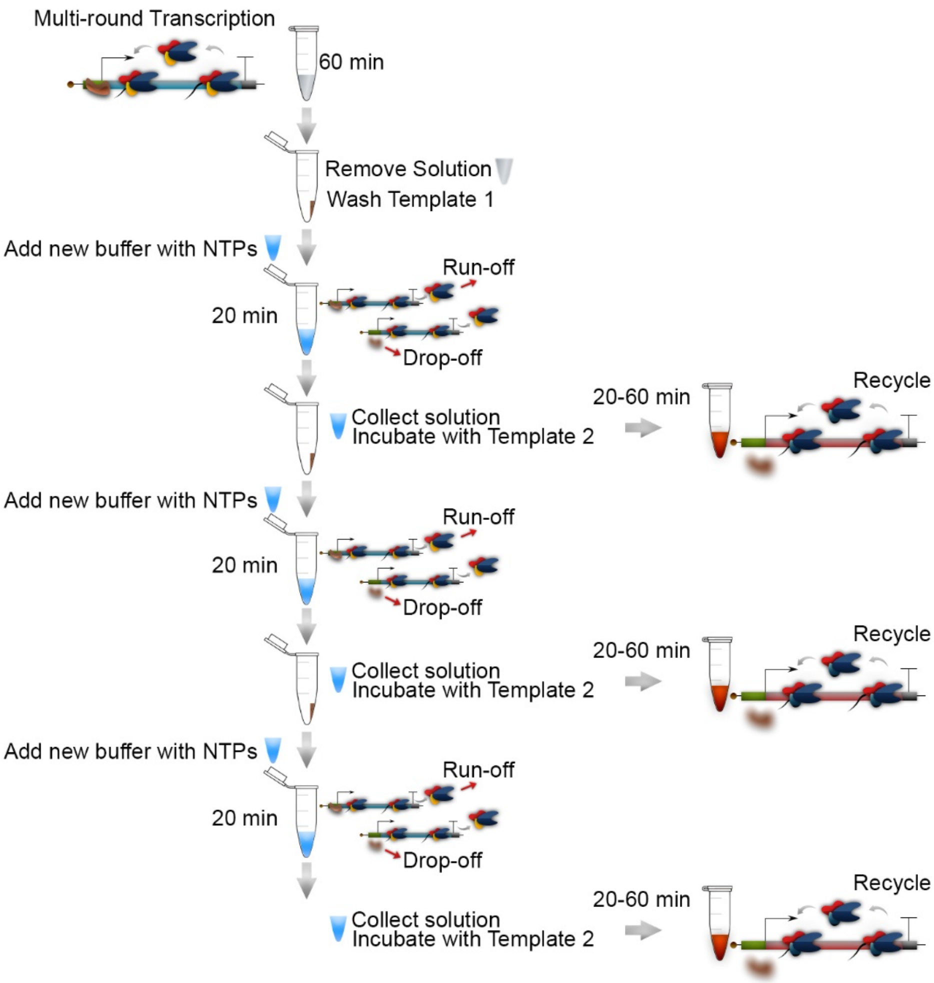

f

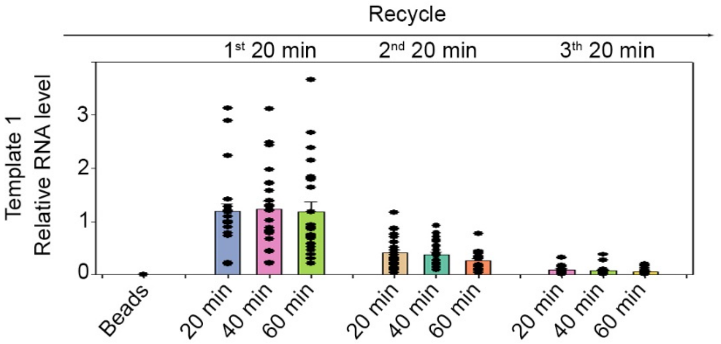

Supplementary Figure 1. ***In vitro*** **transcription recycling system. a, c** The composition of the CMV template and the EF1α template. **b, d** Comparison of RNA products from the full-length CMV and EF1α templates with those from promoter-deleted templates. **e** Schematic of the *in vitro* transcription recycling assay. **f** *In vitro* transcribed 1st template RNA products from the 2nd template during the Recycle step were analyzed by reverse transcription followed by qPCR quantification. Values were expressed as the mean ± SEM of three independent experiments. Source data are provided as a Source data file.

a

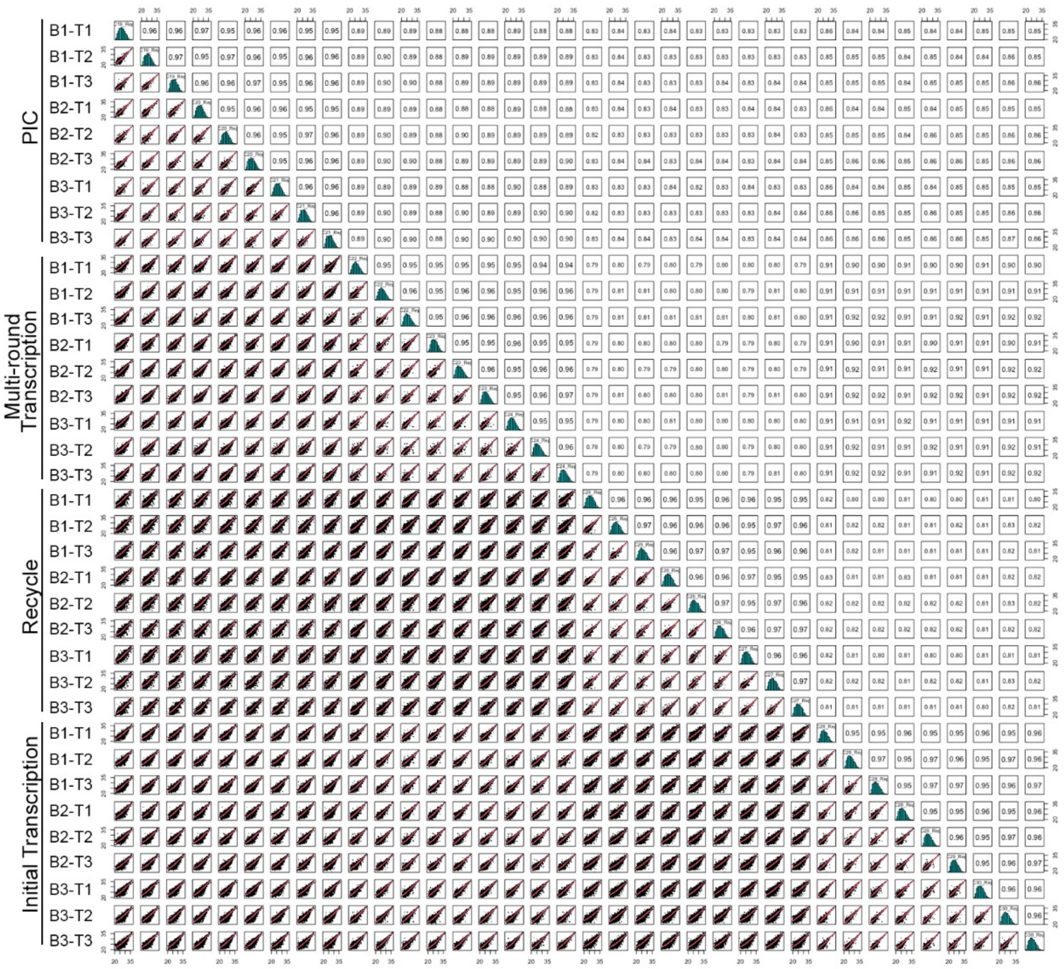

Supplementary Figure 2. Overall analysis and quality control for proteomics data. a Pairwise 2D correlation of protein intensities across all samples.

b

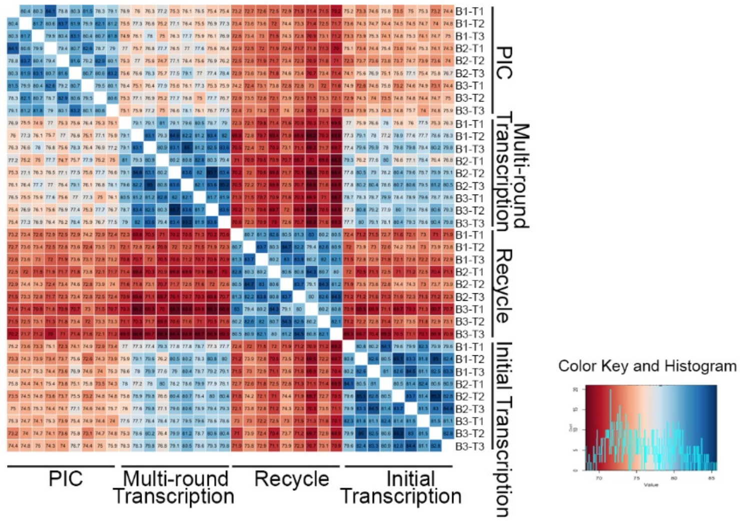

c

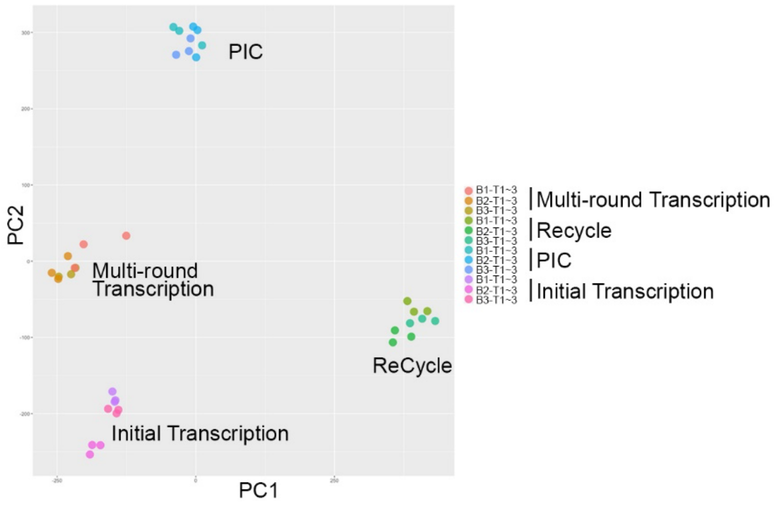

Supplementary Figure 2 (continued). Overall analysis and quality control for proteomics data. b Protein sample-to-sample pairwise identification of overlap among all samples. **c** Principal Component Analysis based on protein intensities across all samples.

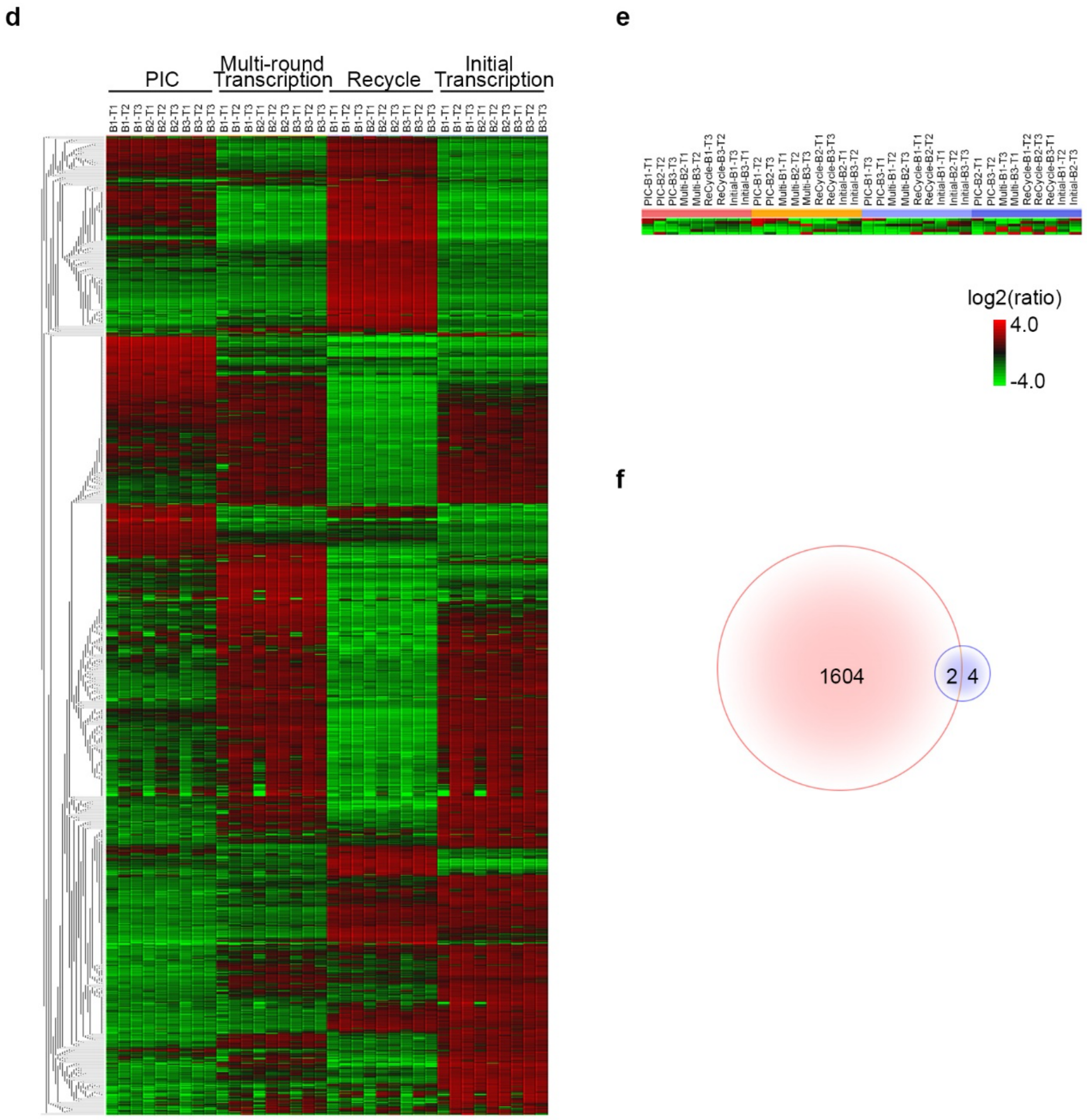

Supplementary Figure 2 (continued). Overall analysis and quality control for proteomics data. d Heatmap of unsupervised clustering showing profiling of proteins identified by LC-MS-MS/MS across all individual transcription steps on the same template. **e** Heatmap of supervised clustering by purposely matching up samples from different conditions. **f** Overlap between proteins represented in both **d** and **e**. Source data are provided as a Source data file.

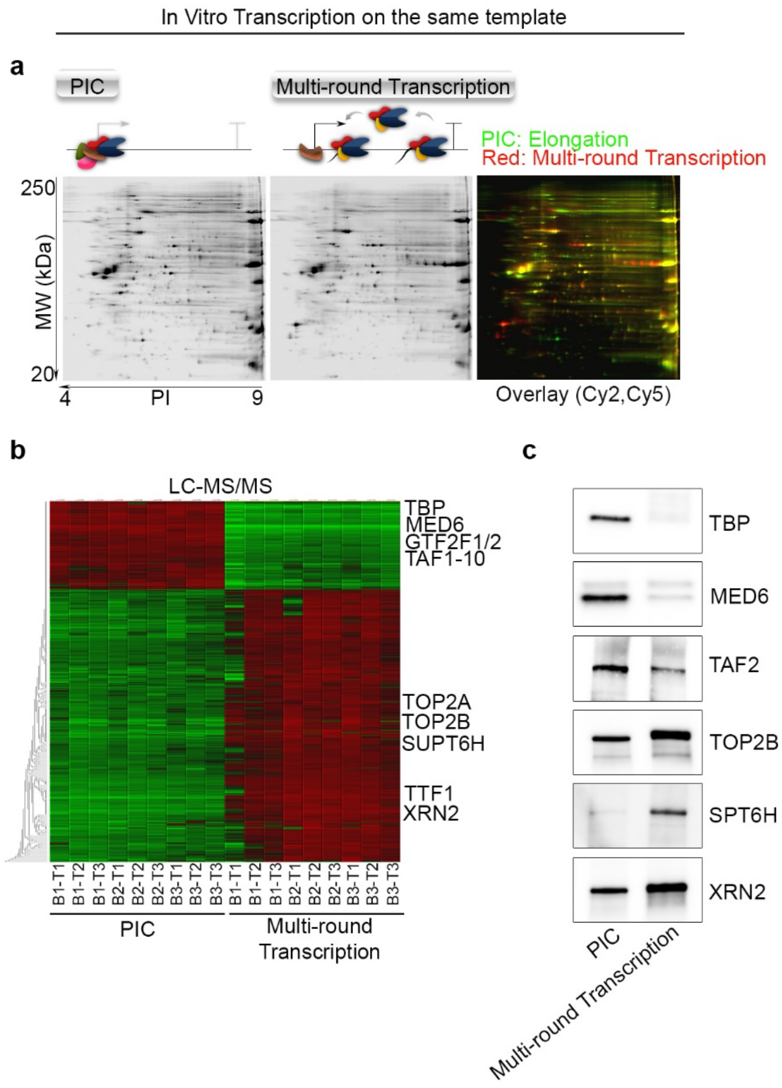

Supplementary Figure 3. Profiling of *in vitro* transcription on the same template. a Representative two-dimensional gels showing proteins bound to the same template in individual transcription steps. **b** Heatmap showing profiling of proteins identified by LC-MS-MS/MS across all individual transcription steps on the same template. Several transcription factors/regulators are labeled. **c** Western blot analysis of proteins bound to the template in individual transcription steps. These images are representative of two independent experiments. Source data are provided as a Source data file.

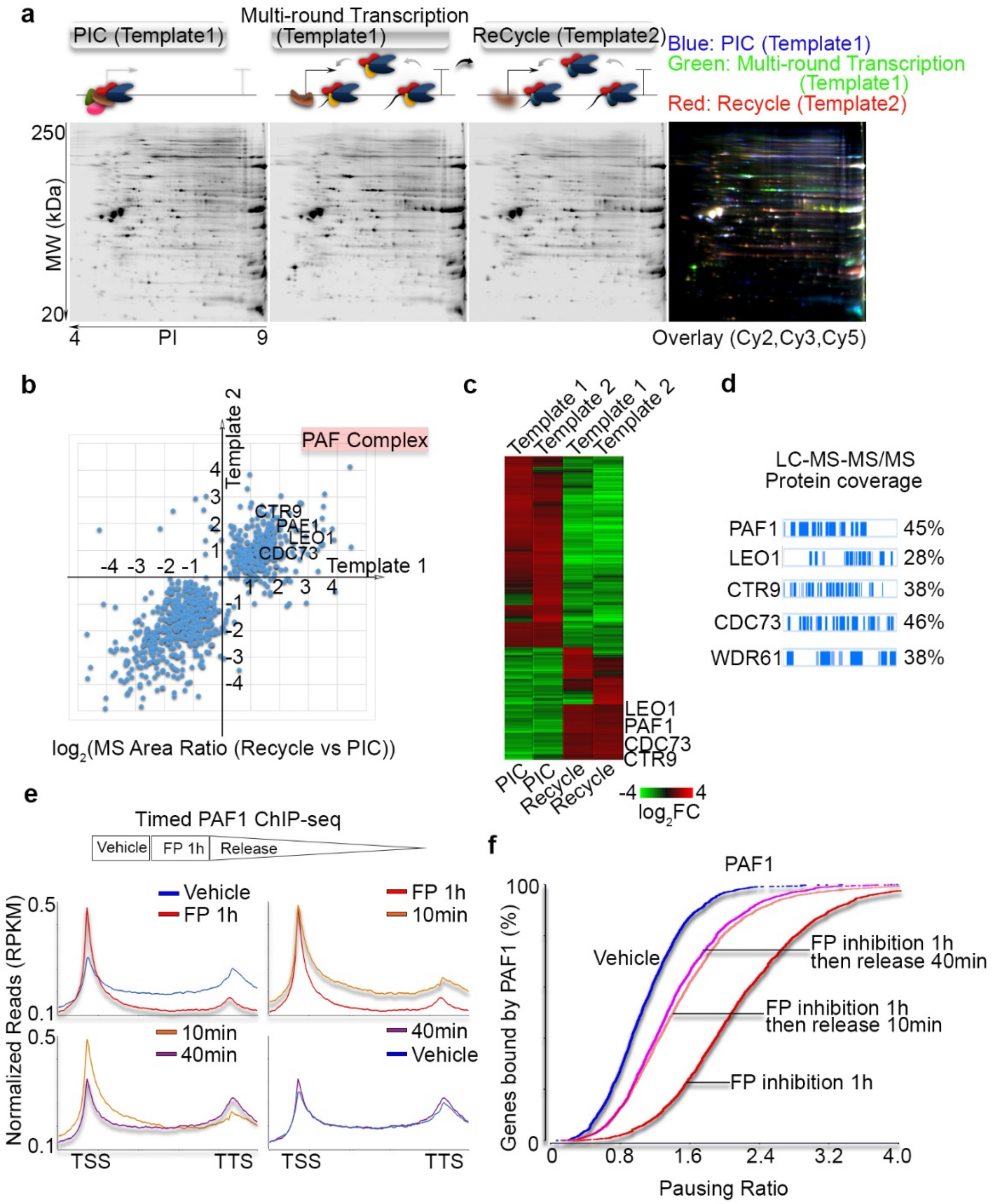

Supplementary Figure 4. Identification and characterization of the PAF1 complex in transcription recycling. **a** Representative two-dimensional gels showing proteins bound to the different sequential templates in individual transcription steps. **b** Comparison of proteins enriched in the Recycle step using two sets of templates. **c** Heatmap showing profiling of proteins identified by LC-MS-MS/MS between the PIC step and Recycle step on two sets of templates. **d** Peptide coverage (highlighted in blue) of PAF1 and additional PAF1C subunits. **e** Average PAF1 ChIP-seq signal densities over the scaled 5000 human RefSeq genes in LNCaP-abl cells. **f** Pausing ratio of PAF1 was calculated, sorted and plotted in LNCaP-abl cells during timed FP inhibition release.

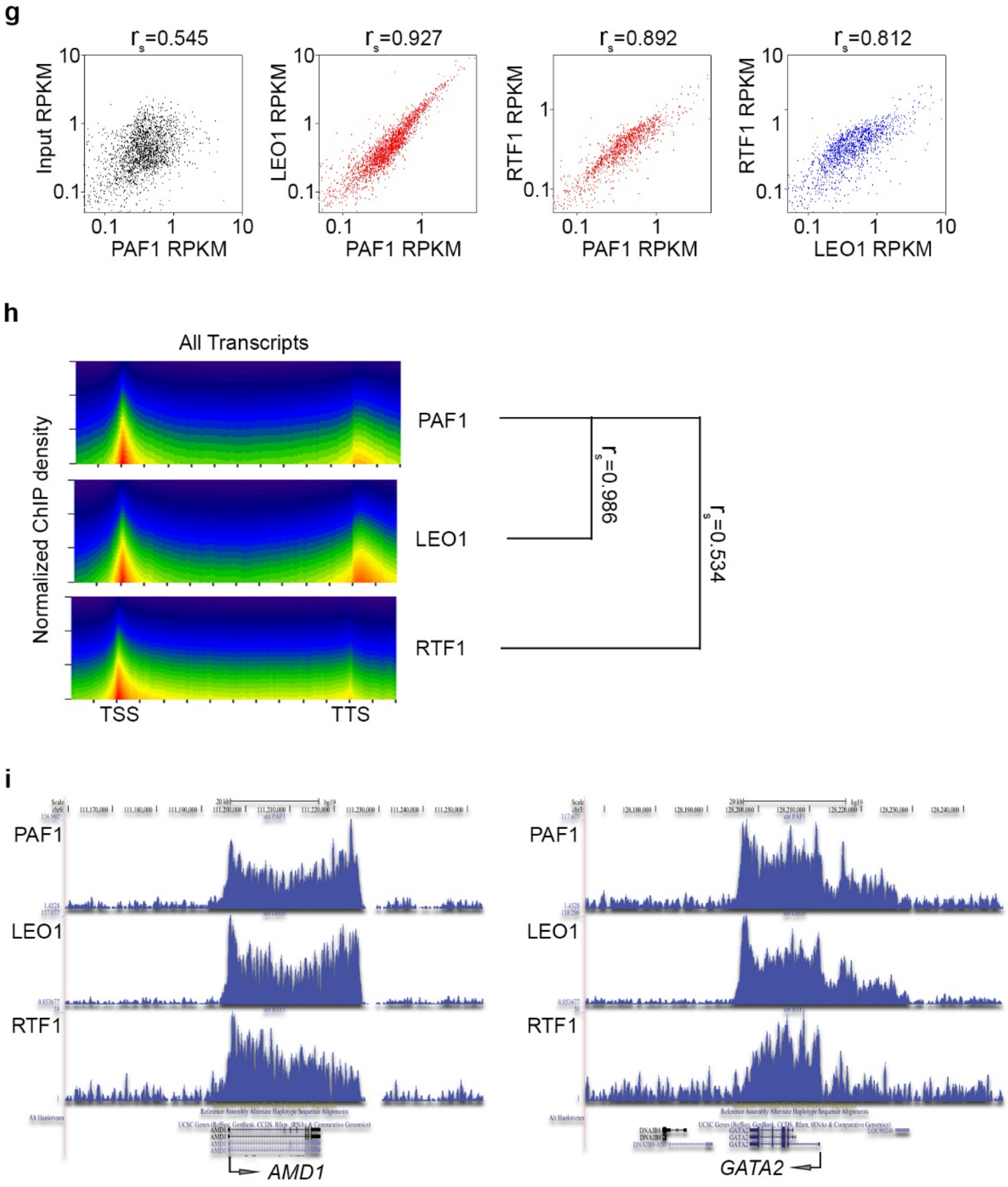

Supplementary Figure 4 (continued). Identification and characterization of the PAF1 complex in transcription recycling. g Correlation between gene body signal density of PAF1, LEO1 and RTF1. **h** Correlation between gene body signal distribution of PAF1, LEO1 and RTF1. **i** UCSC Genome Browser views of representative PAF1, LEO1 and RTF1 co-bindings in LNCaP-abl cells. Source data are provided as a Source data file.

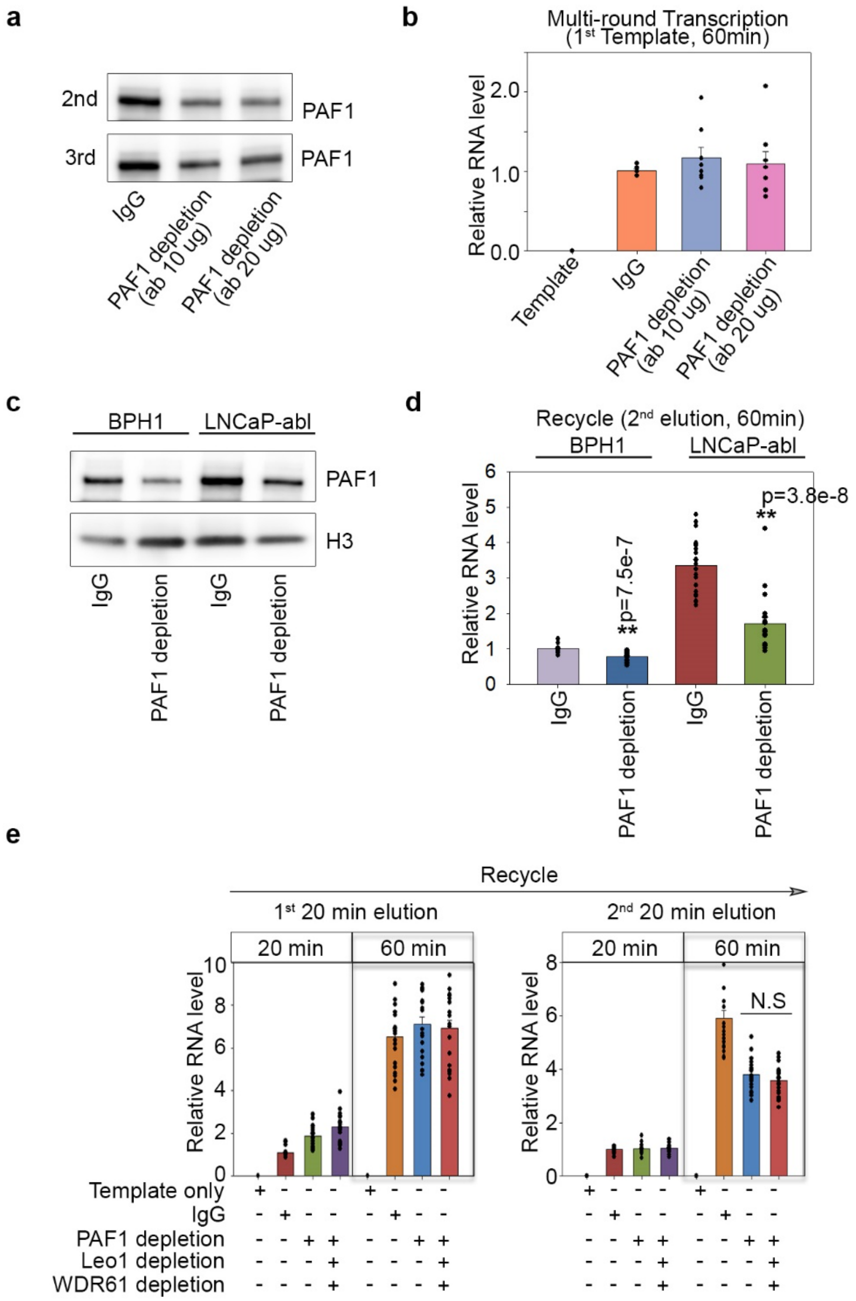

Supplementary Figure 5. PAF1 depletion attenuates transcriptional recycling *in vitro*. a Nuclear extracts from LNCaP-abl cells were incubated with PAF1 antibodies to moderately deplete PAF1 protein, and then were analyzed by western blot. Results were obtained from two additional independent experiments. **b** *In vitro* transcribed 1st template RNA products during the Multi-round Transcription step were analyzed by reverse transcription followed by qPCR quantification. Values were expressed as the mean ± SEM of three independent experiments. **c** Nuclear extracts from BPH1 and LNCaP-abl cells were incubated with PAF1 antibodies to moderately deplete PAF1 protein, and then were analyzed by western blot. These images are representative of two independent experiments. **d** *In vitro* transcribed RNA products

from the 2nd template during the Recycle step were analyzed by reverse transcription followed by qPCR quantification. Values were expressed as the mean ± SEM of three independent experiments. **e** The transcription recycling assay was performed using nuclear extracts with or without PAF1 depletion, or with combined PAF1/LEO1/WDR61 depletion. *In vitro* transcribed RNA products from the 2nd template during the Recycle step were analyzed by reverse transcription followed by qPCR quantification. Values were expressed as the mean ± SEM of two independent experiments. *p*-values were calculated using two-tailed Student's *t*-test. $*p < 0.05$, $**p < 0.01$.

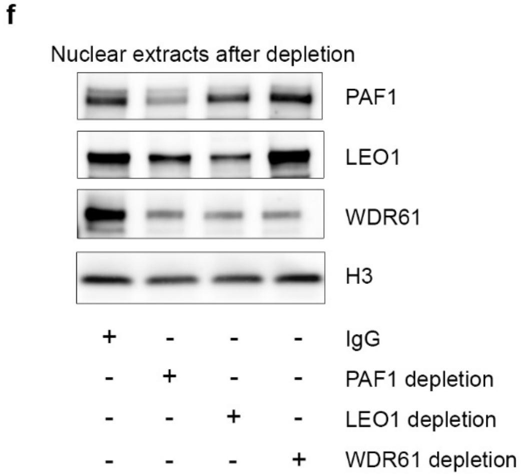

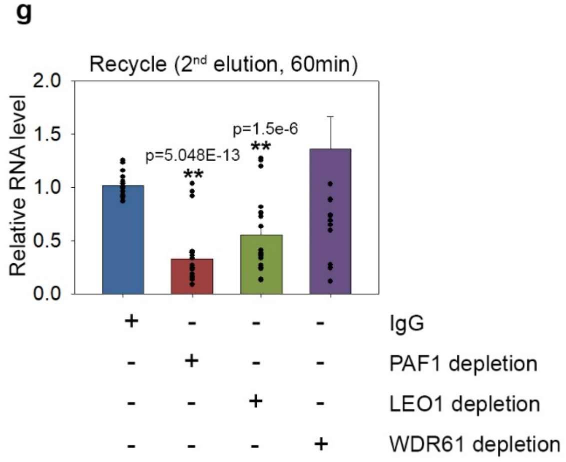

Supplementary Figure 5 (continued). PAF1 depletion attenuates transcriptional recycling *in vitro*. f Nuclear extracts of LNCaP-abl cells were incubated with PAF1, LEO1, or WDR61 antibodies, and then analyzed by western blot. These images are representative of two independent experiments **g** *In vitro* transcribed RNA products from the 2nd template during the Recycle step were analyzed by reverse transcription followed by qPCR quantification. Values were expressed as the mean ± SEM of three independent experiments. *p*-values were calculated using two-tailed Student's *t*-test. $**p < 0.01$. Source data are provided as a Source data file.

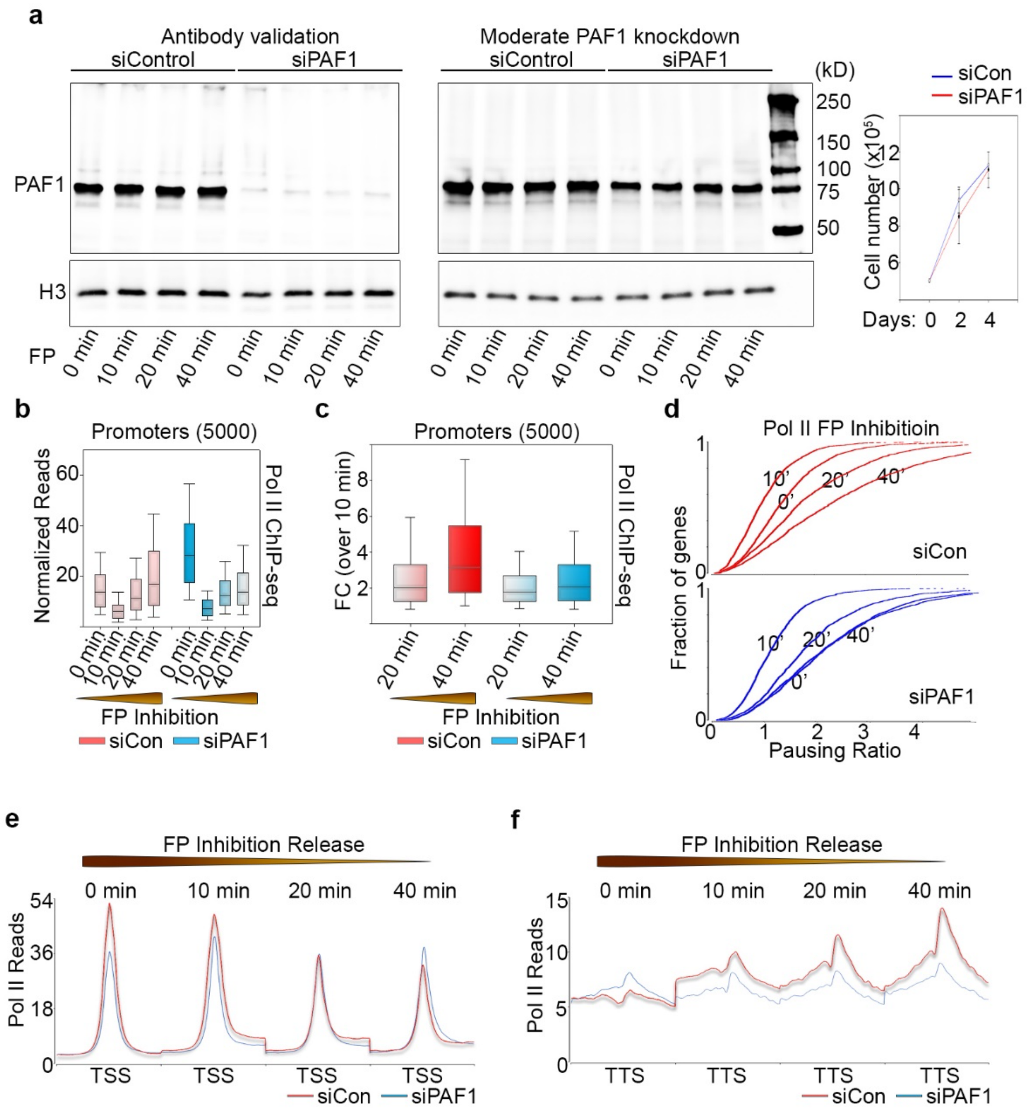

Supplementary Figure 6. PAF1 is required for Pol II transcriptional recycling in human cells. a LNCaP-abl cells were transfected with control or PAF1 siRNA and treated with FP for the indicated time. Protein extracts were then analyzed by immunoblotting to determine PAF1 expression. For antibody validation, 20 nM siRNA was used. For ChIP-seq, 5 nM siRNA was used to accomplish moderate depletion without affecting cell viability. Cell proliferation was measured by direct cell counting assays. Values were expressed as the mean ± SEM of three independent experiments. **b** Box plots show Pol II (Ser2) ChIP-seq signal densities within 5000 promoter regions during timed FP inhibition. Box plots show median (centre), 25th/75th percentiles (box bounds), 10th/90th percentiles (whiskers). The values beyond the whiskers are not shown in the boxplot. **c** Box plots show fold-change in Pol II (Ser2) ChIP-seq signal densities over 10min within 5000 promoter regions during timed FP inhibition. Box plots show median (centre), 25th/75th percentiles (box bounds), 10th/90th percentiles (whiskers). The values beyond the whiskers are not shown in the boxplot. **d** Pausing ratio of Pol II (Ser2) was calculated, sorted and plotted in LNCaP-abl cells with timed FP inhibition. **e** Average Pol II (Ser2) ChIP-seq signal densities over TSS in LNCaP-abl cells during

FP inhibition release. **f** Average Pol II (Ser2) ChIP-seq signal densities over TTS in LNCaP-abl cells during FP inhibition release. Source data are provided as a Source data file.

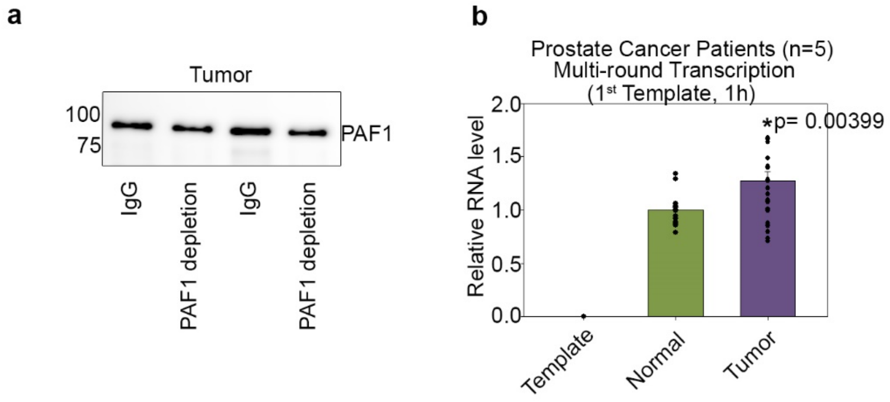

Supplementary Figure 7. Increased transcription activity in tumor tissue. a Nuclear extracts of pooled tissues were incubated with PAF1 antibodies to moderately deplete PAF1 protein, and then were analyzed by western blot. Similar results were obtained from two additional independent experiments. **b** 5 prostate cancer tissues with paired non-tumor adjacent tissues were pooled and used. *In vitro* transcribed 1st template RNA products during the Multi-round Transcription step were analyzed by reverse transcription followed by qPCR quantification. Values were expressed as the mean ± SEM of three independent experiments. *p*-values were calculated using two-tailed Student's *t*-test. *$p < 0.01$. Source data are provided as a Source data file.

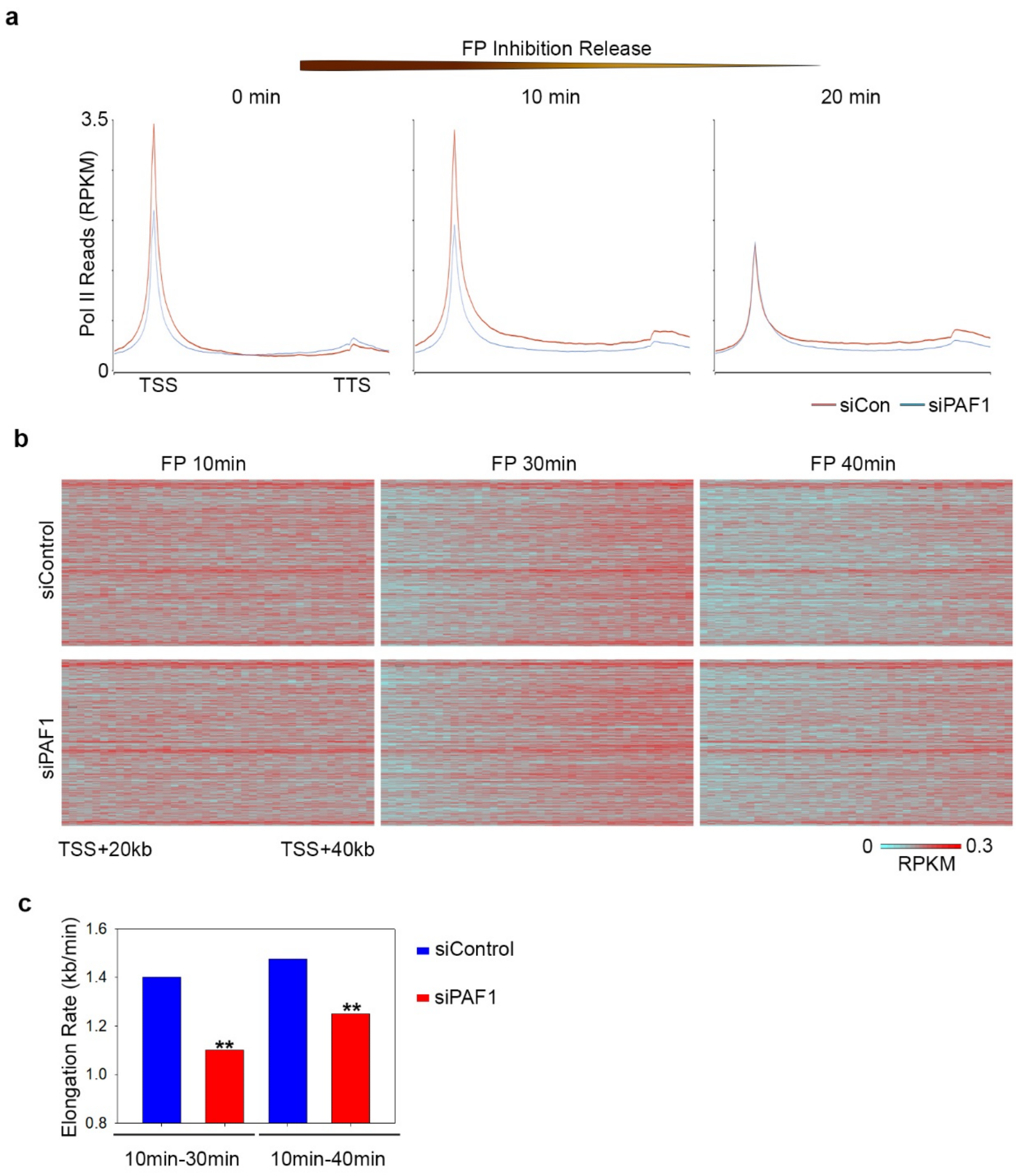

Supplementary Figure 8. Effects of PAF1 silencing on dynamic Pol II binding and transcription elongation rate. a Average Pol II (Ser2) ChIP-seq signal densities over the scaled 5000 human RefSeq genes in LNCaP-abl cells. **b** Heatmap showing normalized raw signal of Pol II (Ser2) ChIP-seq signal over 4600 expressed genes (RPKM>0.3) with gene lengths >70 kb. **c** Elongation rates were calculated using an HMM method. The means of elongation rates were calculated for Pol II (Ser2) ChIP-seq signal at two intervals (10-30min and 10-40min). *p*-values were calculated using two-sided Wilcoxon rank-sum test. ***p* < 0.001. Source data are provided as a Source data file.
