# Supplementary material for: Transcription recycling assays identify PAF1 as a driver for RNA Pol II recycling
Source: Nat Commun. 2021 Nov 3;12:6318. doi: 10.1038/s41467-021-26604-1 (PMC8566496; doi:10.1038/s41467-021-26604-1)
Supplement: Supplementary file 3 — Reporting Summary [file 41467_2021_26604_MOESM3_ESM.pdf]

nature portfolio

Corresponding author(s): Zhong Chen and Qianben Wang

Last updated by author(s): Oct 12, 2021

Reporting Summary

Nature Portfolio wishes to improve the reproducibility of the work that we publish. This form provides structure for consistency and transparency in reporting. For further information on Nature Portfolio policies, see our Editorial Policies and the Editorial Policy Checklist.

Statistics

For all statistical analyses, confirm that the following items are present in the figure legend, table legend, main text, or Methods section.

n/a	Confirmed	
☐	☒	The exact sample size (*n*) for each experimental group/condition, given as a discrete number and unit of measurement
☐	☒	A statement on whether measurements were taken from distinct samples or whether the same sample was measured repeatedly
☐	☒	The statistical test(s) used AND whether they are one- or two-sided<br>*Only common tests should be described solely by name; describe more complex techniques in the Methods section.*
☐	☒	A description of all covariates tested
☐	☒	A description of any assumptions or corrections, such as tests of normality and adjustment for multiple comparisons
☐	☒	A full description of the statistical parameters including central tendency (e.g. means) or other basic estimates (e.g. regression coefficient) AND variation (e.g. standard deviation) or associated estimates of uncertainty (e.g. confidence intervals)
☐	☒	For null hypothesis testing, the test statistic (e.g. *F*, *t*, *r*) with confidence intervals, effect sizes, degrees of freedom and *P* value noted<br>*Give P values as exact values whenever suitable.*
☒	☐	For Bayesian analysis, information on the choice of priors and Markov chain Monte Carlo settings
☐	☒	For hierarchical and complex designs, identification of the appropriate level for tests and full reporting of outcomes
☐	☒	Estimates of effect sizes (e.g. Cohen's *d*, Pearson's *r*), indicating how they were calculated

Our web collection on statistics for biologists contains articles on many of the points above.

Software and code

Policy information about availability of computer code

Data collection	Genomic sequencing data was collected using Hiseq-2500/4000, Nextseq550 and Novaseq 6000 system. Proteomics data was collected using Orbitrap Fusion Lumas platform.
Data analysis	NGS data was analyzed using bowtie2-2.1.0, samtools-1.2, MACS2-2.1.1, HOMER v4.0 and Genomics Suite 7.18. Proteomics data was analyzed by Peak Studio X. AzureSpot Analysis Software 2.0 was used to perform western blot densitometry. Sigmaplot 13.0 was used for statistical analysis.

For manuscripts utilizing custom algorithms or software that are central to the research but not yet described in published literature, software must be made available to editors and reviewers. We strongly encourage code deposition in a community repository (e.g. GitHub). See the Nature Portfolio guidelines for submitting code & software for further information.

Data

Policy information about availability of data

All manuscripts must include a data availability statement. This statement should provide the following information, where applicable:

- Accession codes, unique identifiers, or web links for publicly available datasets
- A description of any restrictions on data availability
- For clinical datasets or third party data, please ensure that the statement adheres to our policy

High throughput sequencing data were deposited in the Gene Expression Omnibus database under accession number GSE133655. Proteomics data were deposited in PRIDE database under accession number PXD027143 and PXD017881.

Field-specific reporting

Please select the one below that is the best fit for your research. If you are not sure, read the appropriate sections before making your selection.

- [x] Life sciences
- [ ] Behavioural & social sciences
- [ ] Ecological, evolutionary & environmental sciences

For a reference copy of the document with all sections, see nature.com/documents/nr-reporting-summary-flat.pdf

Life sciences study design

All studies must disclose on these points even when the disclosure is negative.

Sample size	Sample sizes were chosen according to the standards of the field (at least three independent biological replicates for proteomics, at least two independent biological replicates for high throughput sequencing and other molecular and cellular experiments)
Data exclusions	No data exclusion was performed in the study
Replication	We performed recycling assay three times. For Proteomics data, 9 replicates were examined. For ChIP-seq, 4 replicates were sequenced. All attempts on replication were successful.
Randomization	N/A No animal subjects were used in the study.
Blinding	N/A Investigators and samples were not blinded. Blinding is not used in the field.

Reporting for specific materials, systems and methods

We require information from authors about some types of materials, experimental systems and methods used in many studies. Here, indicate whether each material, system or method listed is relevant to your study. If you are not sure if a list item applies to your research, read the appropriate section before selecting a response.

Materials & experimental systems

n/a	Involved in the study	
[ ]	[x]	Antibodies
[ ]	[x]	Eukaryotic cell lines
[x]	[ ]	Palaeontology and archaeology
[x]	[ ]	Animals and other organisms
[x]	[ ]	Human research participants
[x]	[ ]	Clinical data
[x]	[ ]	Dual use research of concern

Methods

n/a	Involved in the study	
[ ]	[x]	ChIP-seq
[x]	[ ]	Flow cytometry
[x]	[ ]	MRI-based neuroimaging

Antibodies

Antibodies used

Pol II (N-20) Santa Cruz Cat#sc-899
Pol II (8WG16) Abcam Cat#ab817
Pol II (phospho S2) Abcam Cat#ab5095
PAF1 Abcam Cat#ab20662
PAF1 Abcam Cat#ab137519
PAF1 Novus Biologicals Cat#NB600-273
LEO1 Bethyl Laboratories Cat#A300-175A
WDR61 Novus Biologicals Cat#NBP2-59231
WDR61 Novus Biologicals Cat#NBP2-46359
Rtf1 Abcam, Cat#ab99362
H3 Abcam Cat#ab209023
TBP Abcam Cat#ab197874
MED6 Termo Cat# PA5-40846
SPT6H (H-227) Santa Cruz Biotechnology Cat# sc-367017
TOP2B (H-286) Santa Cruz Biotechnology Cat# sc-13059
TAF2 (6B3) Santa Cruz Biotechnology Cat# sc-735
XRN2 (H-300) Santa Cruz Biotechnology Cat# sc-99237
Goat anti-mouse IgG (H+L) secondary antibody, HRP Azure biosystems Cat#AC2115
Goat anti-rabbit IgG (H+L) Secondary antibody, HRP Azure biosystems Cat#AC2114
Donkey anti-goat IgG-HRP Santa Cruz Biotechnology Cat# sc-2020

Validation	The validation of all primary antibodies except for PAF1 antibody for the species and application, noting any validation statements on the manufacturer's website. The validation of PAF1 antibody for the species and application, noting any validation statements or data provided in the manuscript.

Eukaryotic cell lines

Policy information about cell lines

Cell line source(s)	The human prostate cancer cell line abl was provided by Dr. Zoran Culig (Innsbruck Medical University, Austria). The human female HeLa cell line was obtained from ATCC. The human benign prostatic hyperplasia cell line was obtained from Sigma.
Authentication	Cells were authenticated by short tandem repeat (STR) profiling and karyotyping or as described previously (Culig, et al. Br J Cancer 1999;81(2):242-51).
Mycoplasma contamination	The cell lines will be routinely tested to ensure they are free of mycoplasma contamination (VenorTMGeM Mycoplasma Detection Kit, Sigma-Aldrich). All cell lines tested negative for mycoplasma contamination.
Commonly misidentified lines (See ICLAC register)	No commonly misidentified cell lines were used in the study.

ChIP-seq

Data deposition

☒ Confirm that both raw and final processed data have been deposited in a public database such as GEO.

☒ Confirm that you have deposited or provided access to graph files (e.g. BED files) for the called peaks.

Data access links *May remain private before publication.*	https://www.ncbi.nlm.nih.gov/geo/query/acc.cgi?token=cbonqaqkntyfnot&acc=GSE133655
Files in database submission	GSM3914212 siCon-FP1h-0-PAF<br>GSM3914213 siCon-FP1h-10-PAF<br>GSM3914214 siCon-FP1h-20-PAF<br>GSM3914215 siCon-FP1h-30-PAF<br>GSM3914216 siCon-FP1h-40-PAF<br>GSM3914217 siPAF-FP-0-5095<br>GSM3914218 siPAF-FP-10-5095<br>GSM3914219 siPAF-FP1h-0-5095<br>GSM3914220 siPAF-FP1h-10-5095<br>GSM3914221 siPAF-FP1h-20-5095<br>GSM3914222 siPAF-FP1h-30-5095<br>GSM3914223 siPAF-FP1h-40-5095<br>GSM3914224 siPAF-FP-20-5095<br>GSM3914225 siPAF-FP-30-5095<br>GSM3914226 siPAF-FP-40-5095<br>GSM5430140 siCon-FP1h-0-PAF_rep2<br>GSM5430141 siCon-FP1h-0-PAF_rep3<br>GSM5430142 siCon-FP1h-0-PAF_rep4<br>GSM5430143 siCon-FP1h-0-PAF_rep5<br>GSM5430144 siCon-FP1h-10-PAF_rep2<br>GSM5430145 siCon-FP1h-10-PAF_rep3<br>GSM5430146 siCon-FP1h-10-PAF_rep4<br>GSM5430147 siCon-FP1h-10-PAF_rep5<br>GSM5430148 siCon-FP1h-20-PAF_rep2<br>GSM5430149 siCon-FP1h-20-PAF_rep3<br>GSM5430150 siCon-FP1h-20-PAF_rep4<br>GSM5430151 siCon-FP1h-20-PAF_rep5<br>GSM5430152 siCon-FP1h-30-PAF_rep2<br>GSM5430153 siCon-FP1h-30-PAF_rep3<br>GSM5430154 siCon-FP1h-30-PAF_rep4<br>GSM5430155 siCon-FP1h-30-PAF_rep5<br>GSM5430156 siCon-FP1h-40-PAF_rep2<br>GSM5430157 siCon-FP1h-40-PAF_rep3<br>GSM5430158 siCon-FP1h-40-PAF_rep4<br>GSM5430159 siCon-FP1h-40-PAF_rep5<br>GSM5430160 siPAF-FP-0-5095_rep2<br>GSM5430161 siPAF-FP-0-5095_rep3<br>GSM5430162 siPAF-FP-0-5095_rep4<br>GSM5430163 siPAF-FP-0-5095_rep5

GSM5430164 siPAF-FP-10-5095_rep2
GSM5430165 siPAF-FP-10-5095_rep3
GSM5430166 siPAF-FP-10-5095_rep4
GSM5430167 siPAF-FP-10-5095_rep5
GSM5430168 siPAF-FP-20-5095_rep2
GSM5430169 siPAF-FP-20-5095_rep3
GSM5430170 siPAF-FP-20-5095_rep4
GSM5430171 siPAF-FP-20-5095_rep5
GSM5430172 siPAF-FP-30-5095_rep2
GSM5430173 siPAF-FP-30-5095_rep3
GSM5430174 siPAF-FP-30-5095_rep4
GSM5430175 siPAF-FP-30-5095_rep5
GSM5430176 siPAF-FP-40-5095_rep2
GSM5430177 siPAF-FP-40-5095_rep3
GSM5430178 siPAF-FP-40-5095_rep4
GSM5430179 siPAF-FP-40-5095_rep5
GSM5430180 siPAF-FP1h-0-5095_rep2
GSM5430181 siPAF-FP1h-0-5095_rep3
GSM5430182 siPAF-FP1h-0-5095_rep4
GSM5430183 siPAF-FP1h-0-5095_rep5
GSM5430184 siPAF-FP1h-10-5095_rep2
GSM5430185 siPAF-FP1h-10-5095_rep3
GSM5430186 siPAF-FP1h-10-5095_rep4
GSM5430187 siPAF-FP1h-10-5095_rep5
GSM5430188 siPAF-FP1h-20-5095_rep2
GSM5430189 siPAF-FP1h-20-5095_rep3
GSM5430190 siPAF-FP1h-20-5095_rep4
GSM5430191 siPAF-FP1h-20-5095_rep5
GSM5430192 siPAF-FP1h-30-5095_rep2
GSM5430193 siPAF-FP1h-30-5095_rep3
GSM5430194 siPAF-FP1h-30-5095_rep4
GSM5430195 siPAF-FP1h-30-5095_rep5
GSM5430196 siPAF-FP1h-40-5095_rep2
GSM5430197 siPAF-FP1h-40-5095_rep3
GSM5430198 siPAF-FP1h-40-5095_rep4
GSM5430199 siPAF-FP1h-40-5095_rep5
GSM5430200 siCon-FP1h-0-5095_rep2
GSM5430201 siCon-FP1h-0-5095_rep3
GSM5430202 siCon-FP1h-0-5095_rep4
GSM5430203 siCon-FP1h-0-5095_rep5
GSM5430204 siCon-FP1h-10-5095_rep2
GSM5430205 siCon-FP1h-10-5095_rep3
GSM5430206 siCon-FP1h-10-5095_rep4
GSM5430207 siCon-FP1h-10-5095_rep5
GSM5430208 siCon-FP1h-20-5095_rep2
GSM5430209 siCon-FP1h-20-5095_rep3
GSM5430210 siCon-FP1h-20-5095_rep4
GSM5430211 siCon-FP1h-20-5095_rep5
GSM5430212 siCon-FP1h-30-5095_rep2
GSM5430213 siCon-FP1h-30-5095_rep3
GSM5430214 siCon-FP1h-30-5095_rep4
GSM5430215 siCon-FP1h-30-5095_rep5
GSM5430216 siCon-FP1h-40-5095_rep2
GSM5430217 siCon-FP1h-40-5095_rep3
GSM5430218 siCon-FP1h-40-5095_rep4
GSM5430219 siCon-FP1h-40-5095_rep5
GSM5430220 siCon-FP-0-5095_rep2
GSM5430221 siCon-FP-0-5095_rep3
GSM5430222 siCon-FP-0-5095_rep4
GSM5430223 siCon-FP-0-5095_rep5
GSM5430224 siCon-FP-10-5095_rep2
GSM5430225 siCon-FP-10-5095_rep3
GSM5430226 siCon-FP-10-5095_rep4
GSM5430227 siCon-FP-10-5095_rep5
GSM5430228 siCon-FP-20-5095_rep2
GSM5430229 siCon-FP-20-5095_rep3
GSM5430230 siCon-FP-20-5095_rep4
GSM5430231 siCon-FP-20-5095_rep5

GSM5430232 siCon-FP-30-5095_rep2
GSM5430233 siCon-FP-30-5095_rep3
GSM5430234 siCon-FP-30-5095_rep4
GSM5430235 siCon-FP-30-5095_rep5
GSM5430236 siCon-FP-40-5095_rep2
GSM5430237 siCon-FP-40-5095_rep3
GSM5430238 siCon-FP-40-5095_rep4
GSM5430239 siCon-FP-40-5095_rep5
GSM5430240 Abl_LEO1_rep1
GSM5430241 Abl_LEO1_rep2
GSM5430242 Abl_LEO1_rep3
GSM5430243 Abl_LEO1_rep4
GSM5430244 Abl_RTF1_rep1
GSM5430245 Abl_RTF1_rep2
GSM5430246 Abl_RTF1_rep3
GSM5430247 Abl_RTF1_rep4

Genome browser session
(e.g. UCSC)

https://www.ncbi.nlm.nih.gov/geo/download/?acc=GSE133655&format=file&file=GSE133655%5Fsicon%5FFP%5F40%5F5095%5FR1%2Esorted%2Ebedgraph%2Egz
https://www.ncbi.nlm.nih.gov/geo/download/?acc=GSE133655&format=file&file=GSE133655%5Fsicon%5FFP%5F30%5F5095%5FR1%2Esorted%2Ebedgraph%2Egz
https://www.ncbi.nlm.nih.gov/geo/download/?acc=GSE133655&format=file&file=GSE133655%5Fsicon%5FFP%5F20%5F5095%5FR1%2Esorted%2Ebedgraph%2Egz
https://www.ncbi.nlm.nih.gov/geo/download/?acc=GSE133655&format=file&file=GSE133655%5Fsicon%5FFP%5F10%5F5095%5FR1%2Esorted%2Ebedgraph%2Egz
https://www.ncbi.nlm.nih.gov/geo/download/?acc=GSE133655&format=file&file=GSE133655%5Fsicon%5FFP%5F0%5F5095%5FR1%2Esorted%2Ebedgraph%2Egz
https://www.ncbi.nlm.nih.gov/geo/download/?acc=GSE133655&format=file&file=GSE133655%5Fsicon%5FFP1h%5F40%5FPAF%5FR1%2Esorted%2Ebedgraph%2Egz
https://www.ncbi.nlm.nih.gov/geo/download/?acc=GSE133655&format=file&file=GSE133655%5Fsicon%5FFP1h%5F40%5F5095%5FR1%2Esorted%2Ebedgraph%2Egz
https://www.ncbi.nlm.nih.gov/geo/download/?acc=GSE133655&format=file&file=GSE133655%5Fsicon%5FFP1h%5F30%5FPAF%5FR1%2Esorted%2Ebedgraph%2Egz
https://www.ncbi.nlm.nih.gov/geo/download/?acc=GSE133655&format=file&file=GSE133655%5Fsicon%5FFP1h%5F30%5F5095%5FR1%2Esorted%2Ebedgraph%2Egz
https://www.ncbi.nlm.nih.gov/geo/download/?acc=GSE133655&format=file&file=GSE133655%5Fsicon%5FFP1h%5F20%5FPAF%5FR1%2Esorted%2Ebedgraph%2Egz
https://www.ncbi.nlm.nih.gov/geo/download/?acc=GSE133655&format=file&file=GSE133655%5Fsicon%5FFP1h%5F20%5F5095%5FR1%2Esorted%2Ebedgraph%2Egz
https://www.ncbi.nlm.nih.gov/geo/download/?acc=GSE133655&format=file&file=GSE133655%5Fsicon%5FFP1h%5F10%5FPAF%5FR1%2Esorted%2Ebedgraph%2Egz
https://www.ncbi.nlm.nih.gov/geo/download/?acc=GSE133655&format=file&file=GSE133655%5Fsicon%5FFP1h%5F10%5F5095%5FR1%2Esorted%2Ebedgraph%2Egz
https://www.ncbi.nlm.nih.gov/geo/download/?acc=GSE133655&format=file&file=GSE133655%5Fsicon%5FFP1h%5F0%5FPAF%5FR1%2Esorted%2Ebedgraph%2Egz
https://www.ncbi.nlm.nih.gov/geo/download/?acc=GSE133655&format=file&file=GSE133655%5Fsicon%5FFP1h%5F0%5F5095%5FR1%2Esorted%2Ebedgraph%2Egz
https://www.ncbi.nlm.nih.gov/geo/download/?acc=GSE133655&format=file&file=GSE133655%5FsiPAF%5FFP%5F40%5F5095%5FR1%2Esorted%2Ebedgraph%2Egz
https://www.ncbi.nlm.nih.gov/geo/download/?acc=GSE133655&format=file&file=GSE133655%5FsiPAF%5FFP%5F30%5F5095%5FR1%2Esorted%2Ebedgraph%2Egz
https://www.ncbi.nlm.nih.gov/geo/download/?acc=GSE133655&format=file&file=GSE133655%5FsiPAF%5FFP%5F20%5F5095%5FR1%2Esorted%2Ebedgraph%2Egz
https://www.ncbi.nlm.nih.gov/geo/download/?acc=GSE133655&format=file&file=GSE133655%5FsiPAF%5FFP%5F10%5F5095%5FR1%2Esorted%2Ebedgraph%2Egz
https://www.ncbi.nlm.nih.gov/geo/download/?acc=GSE133655&format=file&file=GSE133655%5FsiPAF%5FFP%5F0%5F5095%5FR1%2Esorted%2Ebedgraph%2Egz
https://www.ncbi.nlm.nih.gov/geo/download/?acc=GSE133655&format=file&file=GSE133655%5FsiPAF%5FFP1h%5F40%5F5095%5FR1%2Esorted%2Ebedgraph%2Egz
https://www.ncbi.nlm.nih.gov/geo/download/?acc=GSE133655&format=file&file=GSE133655%5FsiPAF%5FFP1h%5F30%5F5095%5FR1%2Esorted%2Ebedgraph%2Egz
https://www.ncbi.nlm.nih.gov/geo/download/?acc=GSE133655&format=file&file=GSE133655%5FsiPAF%5FFP1h%5F20%5F5095%5FR1%2Esorted%2Ebedgraph%2Egz
https://www.ncbi.nlm.nih.gov/geo/download/?acc=GSE133655&format=file&file=GSE133655%5FsiPAF%5FFP1h%5F10%5F5095%5FR1%2Esorted%2Ebedgraph%2Egz
https://www.ncbi.nlm.nih.gov/geo/download/?acc=GSE133655&format=file&file=GSE133655%5FsiPAF%5FFP1h%5F0%5F5095%5FR1%2Esorted%2Ebedgraph%2Egz
https://www.ncbi.nlm.nih.gov/geo/download/?acc=GSE133655&format=file&file=GSE133655%5FABL%5FLEO1%5FR1%2Esorted%2Ebedgraph%2Egz
https://www.ncbi.nlm.nih.gov/geo/download/?acc=GSE133655&format=file&file=GSE133655%5FABL%5FRTF1%5FR1%2Esorted%2Ebedgraph%2Egz

Methodology

Replicates	We performed experiments using four to five biological replicates.
Sequencing depth	All data are 50-bp single-end. Each data was normalized to the same sequencing depth (50 million).
Antibodies	PAF1 (Abcam, ab20662)<br>phosphorylated RNA pol II (Abcam, phospho S2)<br>leo1 (Bethyl Laboratories, A300-175A)<br>Rtf1 (Abcam, ab99362)
Peak calling parameters	Default parameters were used
Data quality	All peaks are at 1% FDR
Software	NGS data was analyzed using bowtie2-2.1.0, samtools-1.2, MACS2-2.1.1, HOMER v4.0 and Genomics Suite 7.0.
